# Gouty tophus presenting as an anterior cruciate ligament mass in the knee: Case report and brief review of relevant literature

**DOI:** 10.1016/j.ijscr.2021.105920

**Published:** 2021-04-27

**Authors:** Evan Daniel Curd, Kajeandra Ravichandiran, Jihad Abouali

**Affiliations:** a Michael Garron Hospital, Toronto, ON, Canada; b University of Toronto, Toronto, ON, Canada; c Toronto, ON, Canada

**Keywords:** ACL mass, Gout, Pigmented villonodular synovitis, Case report

## Abstract

**Introduction and importance:**

Tophacious gout presenting at the anterior cruciate ligament (ACL) is extremely rare and difficult to differentiate from other intraarticular pathology. This is mainly due to conventional diagnostic tools, such as MRI, producing ambiguous results versus pigmented villonodular synovitis (PVNS) and ganglion cysts.

**Case presentation:**

Here we report an individual in their late-20s with a gouty tophus located at the origin of the ACL in the knee. Urate crystals on the articular cartilage in all three compartments was noted as well as on the synovium. On advanced imaging with an MRI, a large mass was seen anteriorly in the notch surrounding the ACL and posterior cruciate ligament (PCL). The tophus was biopsied and excised arthroscopically with excellent results.

**Clinical discussion:**

An ACL mass in the knee has a very broad differential diagnosis. MRI imaging alone makes it very difficult to differentiate between PVNS and gout tophi yielding a pre-operative diagnostic challenge. Additionally, we review diagnostic challenges faced by other groups with similar cases, as well as their chosen treatment.

**Conclusion:**

Gouty tophi arising from the origin of the ACL are extremely rare and remain difficult to diagnose due to their ambiguous nature in conventional imaging. In this report, we clearly convey the disparity in the diagnostic protocol for this type of intraarticular pathology. Future research should look to develop a superior protocol for identifying these pathologies to improve diagnostic accuracy.

## Highlights

• Atypical mass presenting at the origin of ACL, MRI unable to accurately diagnose.
• Excisional biopsy revealed tophacious gout, 1 of only 4 cases presenting at ACL.
• Multi-disciplinary approach needed in order to achieve a definitive diagnosis.

## 1 Introduction

Intraarticular masses in the knee present a challenge to surgeons due to broad differential diagnosis. Specifically, masses affecting or originating at the anterior cruciate ligament (ACL) are of particular interest. Common pathology include ganglion cysts, synovitis, pannus, gouty tophi and tenosynovial giant cell tumors, such as pigmented villonodular synovitis (PVNS). Ganglion cysts are masses with myxoid matrices most commonly occurring in connective tissue, although these are very rare [1]. Tenosynovial giant cell tumors are also rare, locally aggressive neoplasm of the synovium [2]. Generally, these are associated with joint destruction, inflammation, swelling and pain. Tophacious gout is the most advanced stage of gout, resulting from urate, proteins, inflammatory cells and giant cells to be deposited into the joint [3,4]. Soft tissue masses called tophi can form in the intraarticular space and cause pain or mechanical impingement [3,4]. Beyond this, tophi may become intrusive to the point where they cause locking of the joint [5,6]. Using conventional imaging such as MRI, it is difficult to differentiate between these various pathologies. We herein report a case of an ACL mass with imaging compatible with multiple diagnoses. This case report has been reported in line with the SCARE Criteria [7].

## 2 Case

**Fig. 1:**
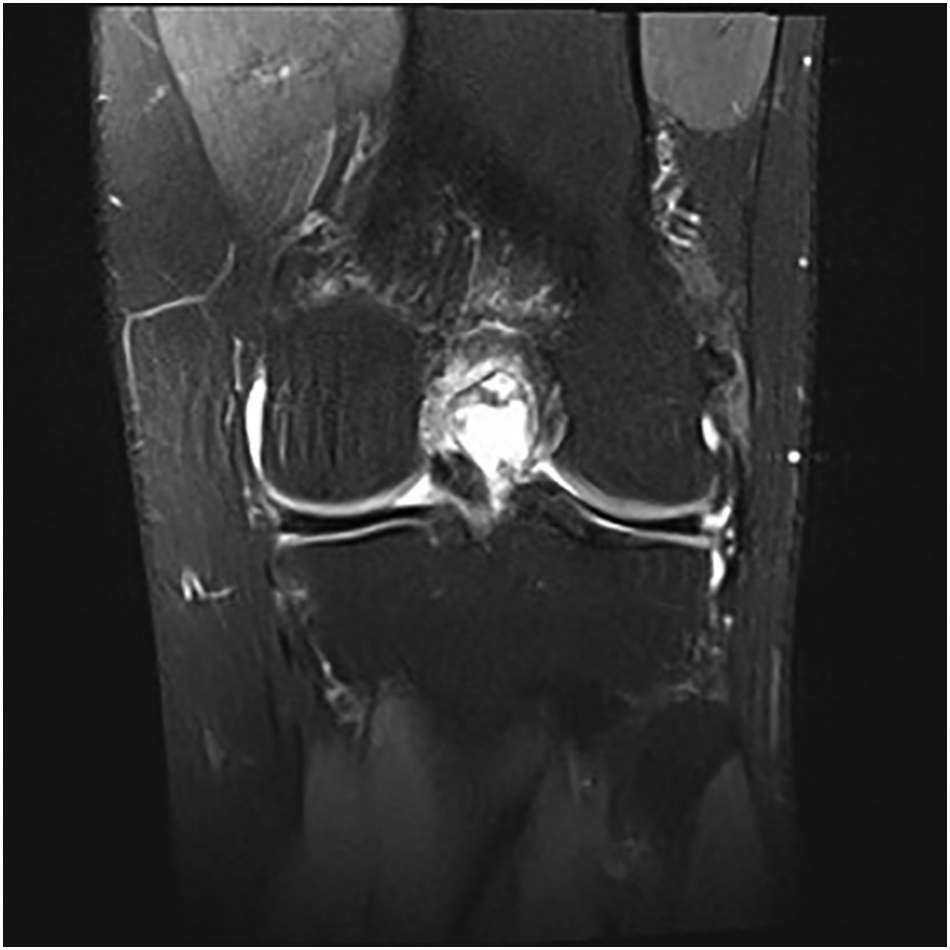
Coronal T3 MRI image (proton density fat saturation) of knee showing large mass filling the notch of the knee and covering the ACL.

**Fig. 2:**
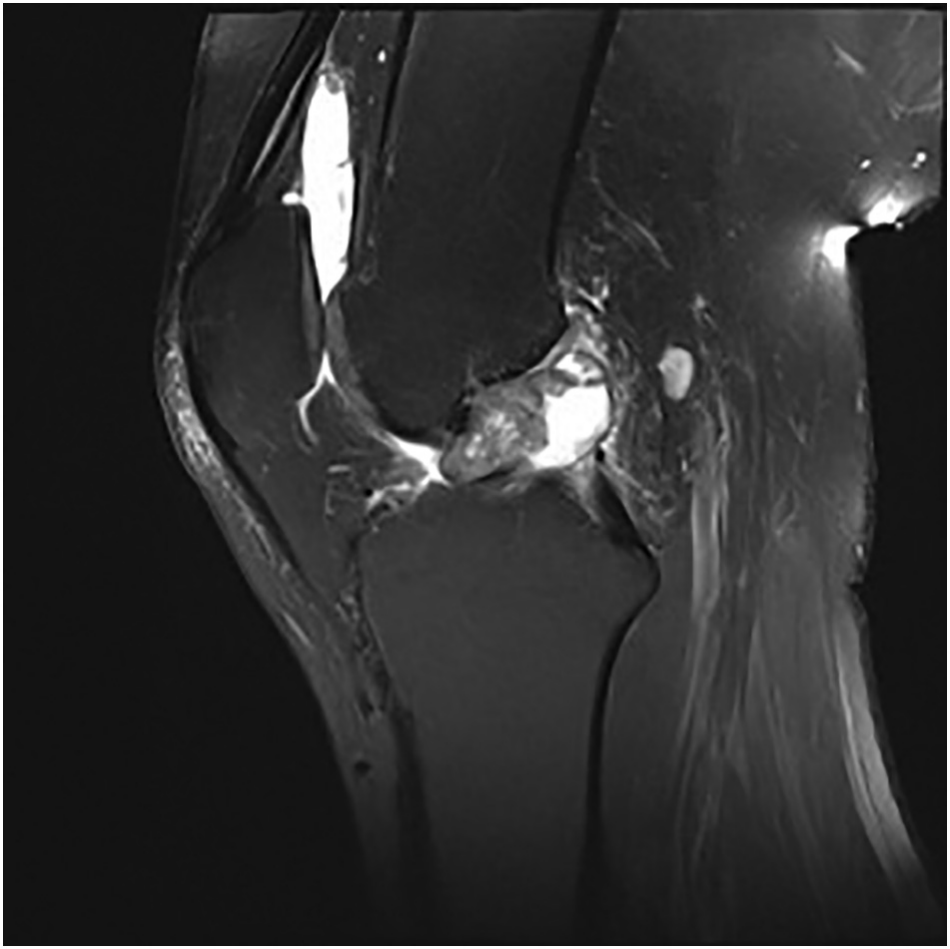
Sagittal T3 MRI image (proton density fat saturation) of knee showing large mass filling the notch of the knee and covering the ACL.

**Fig. 3:**
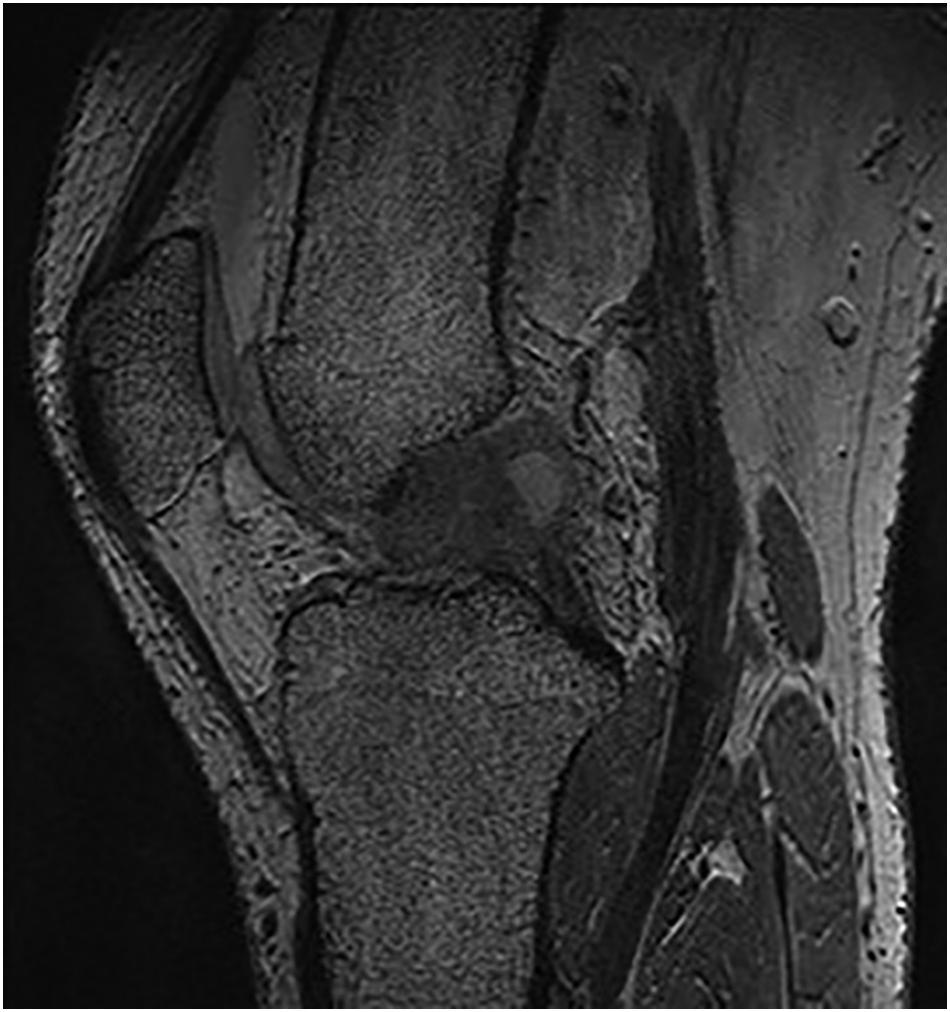
Sagittal T3 MRI image (gradient echo) again revealing a mass within the notch of the knee. Minimal blooming of the lesion is appreciated.

**Fig. 4:**
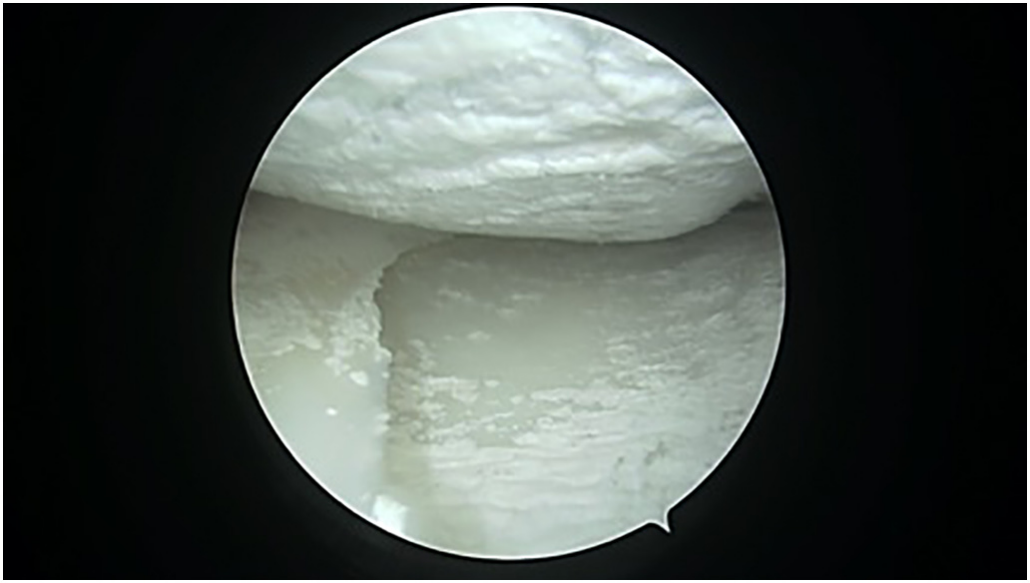
Arthroscopic image of crystalline deposits affecting the entire medial compartment of the knee.

**Fig. 5:**
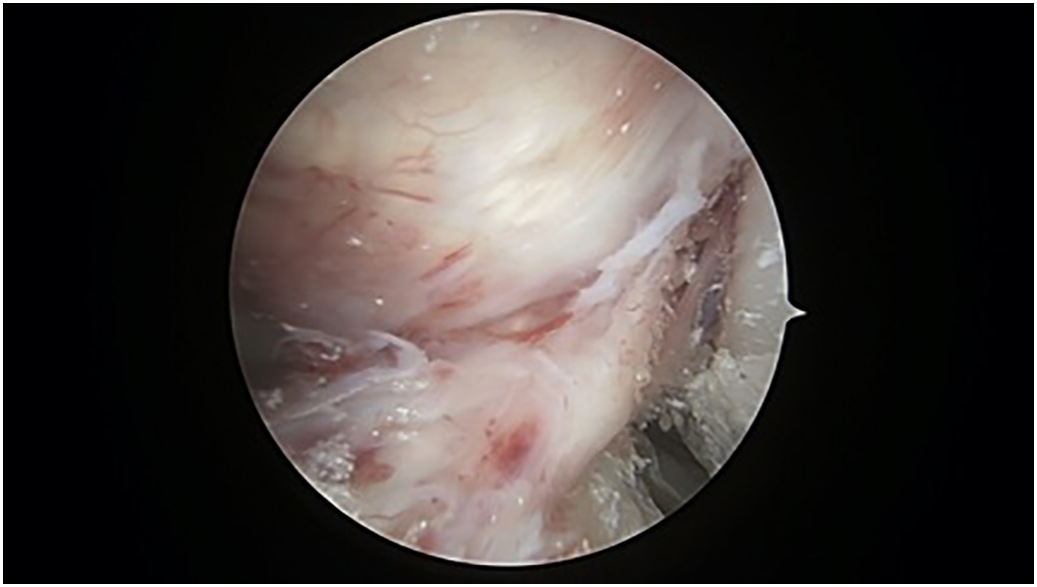
Arthroscopic image showing a large gouty tophus surrounding the ACL.

A 29-year old male, formerly a competitive soccer player, presented with pain in the superior-lateral and posterior aspect of the left knee. He had a history significant for gout that was managed without medications. Physical examination revealed a moderate effusion and an extensor lag of 5 degrees. There was no laxity to the ACL or other ligaments. An initial MRI revealed soft tissue nodularity in the lateral and central aspects of the knee, most consistent with PVNS or gout (Fig. 1, Fig. 2). A second MRI was performed to obtain sagittal gradient echo images on a 3 Tesla MRI unit to confirm the preliminary diagnosis of PVNS. This again revealed the soft-tissue nodularities with a few areas of blooming artifacts, but not to the extent expected for classic PVNS and therefore was thought to be an atypical presentation by our radiology colleagues (Fig. 3). Surgery was scheduled for an arthroscopic knee debridement and excisional biopsy of the lesion by the senior author (JA). During the arthroscopy, significant crystalline formation on the articular cartilage in all three compartments was noted, extending to the synovium (Fig. 4). A large mass was seen anteriorly in the notch surrounding the ACL and PCL (Fig. 5). The mass was resected and sent to pathology for formal analysis. Extrusion of calcified-appearing tissue and crystals from the mass were consistent with a gouty tophus. The mass was debrided in its entirety with care to avoid ACL or PCL injury. The majority of gouty deposits found along the cartilage was debrided carefully with the aid of a shaver to protect the cartilage from further mechanical injury. An extensive synovectomy was also carried out to aid in the reduction of the gout crystal load within the knee. Post-operative histological analysis revealed the excised mass was in fact a gouty tophus. Additionally, since gout was suspected, we performed post-operative uric acid levels which were revealed to be 717 μmol/L. The patient tolerated the procedure well and was ambulating under full weight immediately post-operative. Pain was managed with over-the-counter analgesics. Six- and twelve-month follow-up revealed good range of motion, no effusion and cessation of pain during sporting activities. At two-week follow-up the patient was referred to a rheumatologist for medical management of their gout.

## 3 Discussion

Intraarticular masses involving the ACL are rare [1,2,[8], [9], [10], [11]]. Here we report an extremely rare case of a gouty tophus located at the origin of the ACL. To our knowledge, this is one of only four cases of gouty tophi with origination at the ACL [8,10,11]. There was initial difficulty in coming to a differential diagnosis due to the ambiguous nature of the mass in MRI scans, as gouty tophi and PVNS present similarly using this modality. Specifically for PVNS, the MRI demonstrates joint effusion, hemosiderin deposits, expansion of the synovium and bony erosion [12]. Unfortunately, these characteristics are congruent with gouty tophi in presentation, which further complicate differential diagnosis [13]. The first group to report a gouty tophus originating from the ACL encountered the same difficulties in achieving an accurate pre-operative diagnosis [10]. A pre-operative joint arthrocentesis would have revealed intraarticular monosodium urate crystals, however, it wouldn't have completely ruled out an additional diagnosis. Similar to the present case, three groups reported visualizing uric acid crystals immediately after introducing the arthroscope [8,10,11]. In order to accurately diagnose a gouty tophus, it appears a dual approach using MRI and histological examination is necessary to differentiate it from PVNS and other intraarticular masses [1,2,[8], [9], [10], [11]]. Due to MRI not being a definitive factor in a differential diagnosis, other tests can be utilized. As the present case revealed, gout will manifest under high uric acid levels in the blood [3,8,[10], [11], [12]]. A combination of blood uric acid levels and past history with tophacious gout are strong differentiators for a diagnosis [3,12]. Visualization with the arthroscope also provides key evidence in determining the presence of gout in the synovial space [8,10,11]. In the cases where the ACL mass was gout, the patients had an established prior history [8,10,11]. Interestingly, one group highlighted that their patient's gout had been stable for a significant period of time before becoming symptomatic in the knee. Upon reviewing, this appears to be true in the present and other reviewed cases [8,10,11]. This could be another factor contributing to the difficulty of reaching a diagnosis.

Treatment of ACL masses are fairly uniform throughout the reviewed literature. Most groups approached the mass arthroscopically and debrided to its base until successful excision [8,10,11]. Another group utilized an arthrotomy to visualize and remove the ACL mass [9]. In this case, analysis revealed the mass was a tenosynovial giant cell tumor^,^ and not gout, which may explain the differing approaches [9]. If arthroscopic surgery is not possible or indicated, there are alternatives. One group utilized allopurinol injections in a 67-year-old male with an intraarticular gouty tophi in the knee [14]. Continuous management with allopurinol and narcotics substantially reduced symptoms and resolved the tophi [14].

## 4 Conclusion

Gouty tophi arising from the origin of the ACL are extremely rare and remain difficult to diagnose due to their ambiguous nature in conventional imaging [12,13]. In our case, we found intra-operative arthroscopic visualization of joint crystalline deposits and elevated uric acid level with formal pathological confirmation of the mass necessary in making a diagnosis. The findings of this report add to the growing knowledge base surrounding tophacious gout and its various complications, and aids in the differential of atypical ACL masses. Moving forward, we will suspect an atypically appearing ACL mass to be a gouty tophus in a patient with a high uric acid level. Future investigations into these type of intraarticular pathologies should look to develop a superior protocol for identification and differentiation in order to improve diagnostic accuracy.

## Provenance and peer review

Not commissioned, externally peer-reviewed.

## Sources of funding

None declared.

## Ethical approval

This report is sanctioned by the Michael Garron Hospital REB Committee; ID: NR-303.

## Informed consent

Written informed consent was obtained from the patient for use of their case and medical imaging results. A copy of the written consent may be furnished upon the editors request.

## Research registration

1. Name of the registry: Not applicable.
2. Unique Identifying number or registration ID: Not applicable.
3. Hyperlink to your specific registration (must be publicly accessible and will be checked): Not applicable.

## Guarantor

Jihad Abouali.

## CRediT authorship contribution statement

EC: Drafting and revision. KR: Drafting, revision, approval of final manuscript. JA Drafting, revision, approval of final manuscript.

## Declaration of competing interest

None declared.
